# Conducting Clinical Research During the COVID-19 Pandemic: Investigator and Participant Perspectives

**DOI:** 10.2196/18887

**Published:** 2020-04-06

**Authors:** Prasad R Padala, Ashlyn M Jendro, Kalpana P Padala

**Affiliations:** 1 Geriatric Research Education and Clinical Center Eugene J Towbin Healthcare Center Central Arkansas Veterans Healthcare System North Little Rock, AR United States; 2 Department of Psychiatry University of Arkansas for Medical Sciences Little Rock, AR United States; 3 Department of Geriatrics University of Arkansas for Medical Sciences Little Rock, AR United States

**Keywords:** clinical research, COVID-19, pandemic, outbreak, infectious disease, public health, ethics

## Abstract

As the medical landscape changes daily with the coronavirus disease (COVID-19) pandemic, clinical researchers are caught off-guard and are forced to make decisions on research visits in their ongoing clinical trials. Although there is some guidance from local and national organizations, the principal investigator (PI) is ultimately responsible for determining the risk-benefit ratio of conducting, rescheduling, or cancelling each research visit. The PI should take into consideration the ethical principles of research, local/national guidance, the community risk of the pandemic in their locale, staffing strain, and the risk involved to each participant, to ultimately decide on the course of action. While balancing the rights and protection of the human subject, we seldom examine patients’ views and opinions about their scheduled research visit(s). This article discusses the ethical principles of beneficence and autonomy in helping the decision-making process. We discuss ways to weigh-in local and national guidance, staffing strain, and institutional support into the decision-making process and outline potential changes needed for regulatory bodies depending on the decision. Further, we discuss the need to weigh-in the individual risk-benefit ratio for each participant and present a decision tree to navigate this complex process. Finally, we examine participant and caregiver perspectives on their fears, sense of preparedness, and factors that they consider before deciding whether to keep or postpone the research appointments. This entry also provides PIs ways to support their research participants in both scenarios, including provision of psychological support.

## Introduction

As the medical landscape changes daily with the coronavirus disease (COVID-19) pandemic, clinical researchers are caught off-guard and must make tough decisions about research visits in ongoing clinical trials. Although there is some guidance from local and national organizations, the principal investigator (PI) is ultimately responsible for determining the risk-benefit ratio of conducting, rescheduling, or cancelling each visit. The PI should take into consideration the ethical principles, local/national guidance, the community risk of the pandemic in their locale, staffing strain, and the risk involved to each participant, to ultimately decide the course of action. While balancing the rights and protection of the human subjects, we seldom examine patients’ views and opinions. Here, we present patient perspectives from active research participants (N=51) along with other important considerations to inform the decision-making processes.

## Visit-Related Factors

The fundamental question is whether the research visit changes the risk-benefit ratio discussed in the consent. The ethical principles of beneficence and autonomy should help the PI do what is best for the participants while discussing with each participant about the risk of exposure and the best available knowledge, in order to facilitate their self-determination. At minimum, each participant should be made aware that COVID-19 is now being transmitted from human to human, with a transmissibility rate of 4 [1]. It is particularly infectious due to asymptomatic transmission and symptoms akin to influenza. Ideally, a phone call should be conducted to update the research participant on the current information, screen them for COVID-19, and reassess the risk benefit.

The risk of contagion may vary based on the setting of the research facility. A tertiary care research facility wherein patients with COVID-19 are actively being quarantined or treated, may be at a higher risk than a standalone private research facility. Research participants are at increased risk for COVID-19 infection if they have any comorbidities. Immunocompromised people, pregnant women, and older adults with multiple comorbidities may be particularly vulnerable to serious sequelae [1]. If the risk of contagion or sequela is high, all measures need to be taken to protect the participant. Discussion should also include the risks associated with delay or discontinuation of the study interventions including monitoring, investigational product, and psychological support (if applicable).

## Policy-Related Factors

Local guidance is usually informed by national guidance from the Centers for Disease Control and Prevention (CDC), the National Institutes of Health (NIH), and the Office of Research and Development (ORD). The NIH is aware of the potential disruptions to research and has several directives to guide PIs while placing highest priority on ensuring safety of all participants and allowing for delays in reaching milestones [2]. CDC guidance will help determine the screening process for participants that choose to keep their appointments [3]. The research institution might implement added screening based on the risk of contagion. Such additional screening will need to be conveyed to the participant ahead of time in order to make the best-informed decision about the appointment. If the research institution deems that all research visits need to be halted, such information will need to be communicated to the participant as soon as possible, and alternate arrangements to deliver the intervention/investigational product need to be made.

## Workforce-Related Issues

PIs will need to monitor the staffing of their facility due to sickness or assignment to COVID-19–related tasks. Many institutions are mandating daily screening of their staff with the COVID-19 travel screen. This may add responsibilities to the research staff, necessitating streamlining of the research appointments. Research staff may be concerned about the added risk of infection during in-person visits. Making appropriate information and counseling available could help allay some of these worries. It may be best to prioritize the outcome measures to be collected at each visit, paying particular attention to visits/measures that can be collected over the phone, telehealth, or video chats.

## Investigator Perspective

PIs are best qualified to determine whether their studies can be safely continued, continued with modifications, or temporarily halted. PIs should anticipate disruptions to the study and inform the sponsors and regulatory bodies promptly. When appropriate, they should consider revising their protocol to allow data collection and interaction without in-person contact using phone or videoconference apps such as Skype. Some protocols may already have flexibility regarding visits; otherwise, modification to the protocol may be needed. Informing the institutional review board that the modifications are being made to adapt to COVID-19 may help expedite the review process.

## Research Participant Perspective

Caregiver and participant perspectives are often missed when conducting clinical research during pandemics. Gobat et al [4] reported that 82% of the participants (N=6804) believed it was important to conduct medical research during epidemics. The authors concluded that greater knowledge about pandemics, trust in a health professional, and trust in the government predicted increase in willingness to participate in research. In our convenient sample of 51 informants scheduled for ongoing clinical research studies over a period of 2 weeks on increased surveillance for COVID-19, most felt safe attending the scheduled research appointment (40 reported feeling safe and provided a rating of ≥4 on a Likert scale of 1-5). They also felt that the medical center was well prepared and expressed that the additional screening put them at ease.

Trust in the health care system and the fact that the visit was not in a group format were some of the positive factors reported by the patients in their decision to come for their scheduled appointment. News channels and close family members and friends were the resources that participants most commonly reached out to for decision making. One informant reported to have signed up for the CDC newsletter, while another completely relied on Rush Limbaugh radio coverage. Several participants expressed concern that social media may be contributing to the spread of unauthenticated information and that the public should turn to experts. Informants reported that the general public was in panic about COVID-19 (rating of 4.47 on a Likert scale from 1-5), while some felt that the concern was excessive:

I don’t think it is as serious as people are making it to be.

## Preparation for Distant Visits

If participants or their study partners are not able to come into your site for scheduled visits or a determination has been made for offline visit, have assessments that can be collected by phone or online. Out-of-window visits because of COVID-19 or safety precautions may lead to minor protocol deviations but may not lead to required discontinuations from the study. Institutional and sponsor policies will need to be followed regarding protocol deviation reporting. One positive outcome of the COVID-19 pandemic has been the shift in attitude of the regulatory bodies toward the use of telehealth. Clinical researchers can play a significant role in helping institutional review boards with approval of the use of mobile apps, Skype, Facetime, and other remote platforms to conduct research visits.

## Preparation for In-Person Visits

If the participant or their caregiver decides to come in, make sure that your team is well prepared to handle the visit; for example, avoid any group interactions, provide private rooms for interviews, sanitize the high-traffic and high-touch areas well, minimize contact with the participant, sanitize reusable medical devices per standard operating procedures, and do not share pens for signing forms. Have protective gear such as masks and hand sanitizers ready for both the participants and staff members.

## Addressing Psychological Needs During the Pandemic

Research staff and clinicians have a unique opportunity to address psychological stress due to their ongoing relationship with the participants. A variety of negative psychological effects including posttraumatic stress symptoms, confusion, and anger have been reported as consequences of quarantine [5]. Worry about their family members contracting COVID-19 is a huge concern [1]. Take extra time to address any questions that the participant or their caregiver might have. Assess for any undue mental stress that they might be undergoing and make sure that you have resources or referral services available. Encourage them to be informed about COVID-19 while monitoring that they are not overly exposed. It may be best to limit the checking in to once or twice a day, just enough to take action. Give them practical tips on handling the disruption in their work life by planned breaks during the day, if they are working from home, and leave them with hope that normal processes will resume once the pandemic subsides. Older adults should be screened for loneliness and isolation, an important contributor to all-cause mortality in this age group [6]. Researchers could help older adult participants with tips on ways to stay connected with family and support groups remotely.

## Conclusions

**Figure 1:**
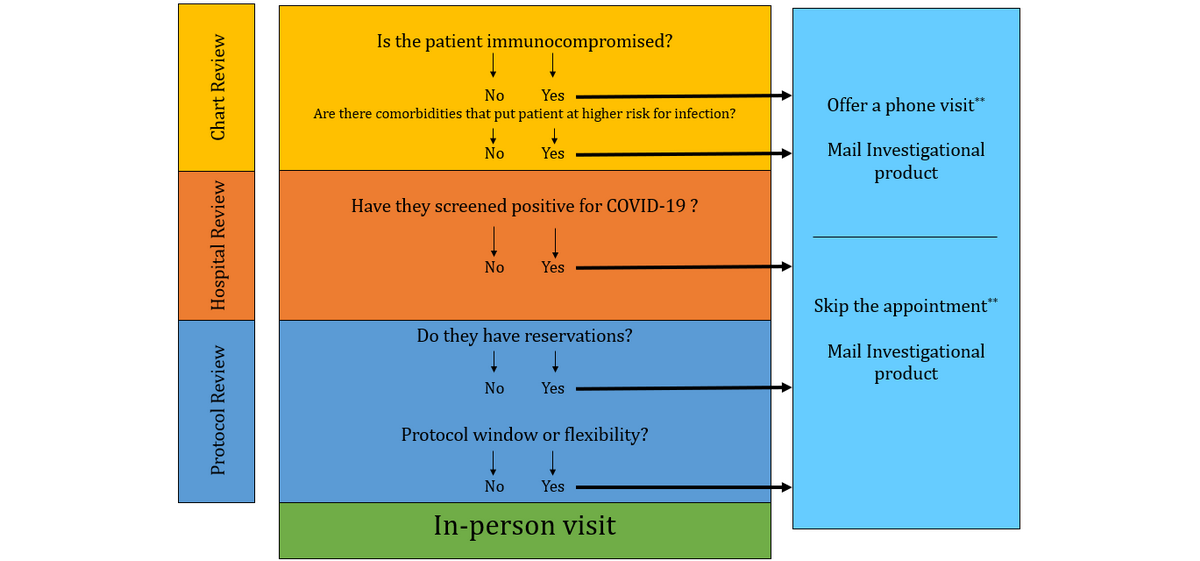
Decision tree for research visits*. COVID-19: coronavirus disease. *Check the local and national guidelines periodically, as the information is changing rapidly. **May need institutional review board approval unless such contingency was built into the protocol. Protocol deviation could be used to take care of the subject, and a modification may need to be applied if you anticipate this to be a recurring issue.

There is no question that clinical researchers are having to make tough decisions about ongoing clinical trials due to the widespread COVID-19 pandemic. Although some guidance has been offered from local and national organizations, it is still ultimately the responsibility of the PI to evaluate the risk-benefit ratio of ongoing research. When making research decisions, PIs should consider all factors that affect the risk-benefit ratio of continuing research during this time. Balancing visit- and policy-related factors as well as the possible lack of a workforce with the perspectives of the research participants can help PIs identify various courses of actions for continued research. Figure 1 presents a decision tree to assist PIs in this decision-making process based off of national recommendations. If PIs chose to and are able to continue clinical research, preparation should be considered in various degrees. PIs’ ability to provide participants with the latest information about COVID-19, provision of a safe environment, and preparedness to address psychological needs will help reassure participants that you have made the most informed decision to continue research.

## Abbreviations

CDC: Center for Disease Control and Prevention
COVID-19: coronavirus disease
NIH: National Institutes of Health
ORD: Office of Research and Development
PI: Principal Investigator
